# Dietary supplementation of recombinant antimicrobial peptide *Epinephelus lanceolatus* piscidin improves growth performance and immune response in *Gallus gallus domesticus*

**DOI:** 10.1371/journal.pone.0230021

**Published:** 2020-03-11

**Authors:** Hsueh-Ming Tai, Han-Ning Huang, Tsung-Yu Tsai, Ming-Feng You, Hung-Yi Wu, Venugopal Rajanbabu, Hsiao-Yun Chang, Chieh-Yu Pan, Jyh-Yih Chen

**Affiliations:** 1 Marine Research Station, Institute of Cellular and Organismic Biology, Academia Sinica, Ilan, Taiwan; 2 Department of Veterinary Medicine, College of Veterinary Medicine, National Pingtung University of Science and Technology, Neipu, Taiwan; 3 Anbil Dharmalingam Agricultural College and Research Institute, Tamil Nadu Agricultural university, Tiruchchirapalli, Tamil Nadu, India; 4 Biotechnology Department, Asia University, Wufeng, Taichung, Taiwan; 5 Department and Graduate Institute of Aquaculture, National Kaohsiung University of Science and Technology, Kaohsiung, Taiwan; 6 The iEGG and Animal Biotechnology Center, National Chung Hsing University, Taichung, Taiwan; National Cheng Kung University, TAIWAN

## Abstract

Supplementing chicken feed with antibiotics can improve survival and prevent disease outbreaks. However, overuse of antibiotics may promote the development of antibiotic-resistant bacteria. Recently, antimicrobial peptides have been proposed as alternatives to antibiotics in animal husbandry. Here, we evaluate the effects of antimicrobial peptide, *Epinephelus lanceolatus* piscidin (EP), in *Gallus gallus domesticus*. The gene encoding EP was isolated, sequenced, codon-optimized and cloned into a *Pichia pastoris* recombinant protein expression system. The expressed recombinant EP (rEP) was then used as a dietary supplement for *G*. *g*. *domesticus*; overall health, growth performance and immunity were assessed. Supernatant from rEP-expressing yeast showed *in vitro* antimicrobial activity against Gram-positive and Gram-negative bacteria, according to an inhibition-zone diameter (mm) assay. Moreover, the antimicrobial peptide function of rEP was temperature independent. The fermentation broth yielded a spray-dried powder formulation containing 262.9 μg EP/g powder, and LC-MS/MS (tandem MS) analysis confirmed that rEP had a molecular weight of 4279 Da, as expected for the 34-amino acid peptide; the DNA sequence of the expression vector was also validated. We then evaluated rEP as a feed additive for *G*. *g*. *domesticus*. Treatment groups included control, basal diet and rEP at different doses (0.75, 1.5, 3.0, 6.0 and 12%). Compared to control, rEP supplementation increased *G*. *g*. *domesticus* weight gain, feed efficiency, IL-10 and IFN-γ production. Our results suggest that crude rEP could provide an alternative to traditional antibiotic feed additives for *G*. *g*. *domesticus*, serving to enhance growth and health of the animals.

## 1. Introduction

Farmed chickens (*Gallus gallus domesticus*) are often fed with antibiotics to promote growth. The antibiotics inhibit or kill pathogens that may negatively affect the health of broilers in order to allow maximal growth. Breeders often pursue methods to increase abundance, consistency, and growth speed of the animals. Therefore, control of disease outbreaks by routine administration of antibiotics is a common practice. For many years, antibiotics have been widely used in poultry farming to reduce bacterial infections, as chicken metabolic responses may occur along with shifts in microbiota composition upon infection [1]. However, antibiotic treatments at certain stages of life can severely discompose the microbiota of the intestine, leading to delays in immune system development and disturbances in immune function. As a result, inappropriate antibiotic treatment can cause the broilers to become susceptible to pathogen infections at later developmental stages [2]. Furthermore, the overuse of antibiotics promotes antibiotic resistance in pathogens, which may then negatively impact animal and human health (https://www.danmap.org/downloads/reports.aspx) [3].

The use of antibiotics for promotion of growth has been banned in Europe since 2006 [4, 5]. Accordingly, new antimicrobial agents that may substitute for antibiotics in poultry production are in demand for use as alternative feed additives. Recently, antimicrobial peptides (AMPs) have emerged as antibiotic equivalents by virtue of their ability to disrupt membrane integrity in bacteria and other pathogens [6]. AMPs exhibit powerful antimicrobial activity against different and diverse microorganisms, but typically still have low hemolytic activity toward host cells [7]. Piscidins comprise one of the most widely studied AMP families. Isolated from several fish species, piscidins exhibit an expansive spectrum of biological functions, including antibacterial, antifungal, anti-parasite, anti-nociceptive and antitumor functions [8–12]. Previously, we cloned and characterized five piscidin-like AMPs (named TP1-TP5) from *Oreochromis niloticus* [9]. Our research on these molecules revealed that pathogens are less able to develop resistance to picidins than to antibiotics. This continued toxicity may be due to the relative non-specificity of electrostatic interactions between piscidins and membrane lipid components of pathogens, such as *Klebsiella pneumoniae* and *Acinetobacter baumannii* [13]. Therefore, pisicidins have potential to take the place of antibiotics as feed additives in the domesticated fowl farming industry.

There are few reports that have described the use of recombinant protein expression systems to produce piscidins [14, 15]. Since piscidins exhibit high antimicrobial activity, the amount of recombinant protein expression tolerated by bacterial hosts may be less than what is necessary for large-scale production of a feed additive. In this study, we found that a crude extract of culture supernatant from recombinant *Epinephelus lanceolatus* piscidin (rEP)-expressing *Pichia pastoris* can be successfully used as a feed additive in livestock products. We rigorously evaluated the effects of dietary supplementation with crude rEP on growth performance and immune response of *G*. *g*. *domesticus*. Furthermore, we characterized the antimicrobial activity of rEP against different pathogens.

## 2. Methods

### 2.1. Animals

All procedures involving animals were conducted in accordance with the requirements of National Pingtung University of Science and Technology (NPUST), and were approved by the Animal Care and Use Committee of NPUST (NPUST-107-026).

### 2.2. Molecular cloning, construction and transformation of expression plasmid, and selection of positive transformants

The sequences of primers used to amplify EP were designed according to a previous transcriptome analysis and experimental method [9, 16]. Briefly, piscidins were isolated from *E*. *lanceolatus* by RT-PCR. mRNA was extracted from *E*. *lanceolatus* liver and reverse transcribed; primers used to amplify cDNA are listed in Table 1. Three recombinant clones (g6496.t1, g6497.t1, g6498.t1) were chosen for sequencing. Multiple sequence alignments of the clones were made with *E*. *lanceolatus* and *O*. *niloticus* piscidin peptides. The g6496.t1 (Ac-CIMKHLRNLWNGAKAIYNGAKAGWTEFK-NH2), g6497.t1 (Ac-CFFRHIKSFWRGAKAIFRGARQGWRE-NH2) and g6498.t1 (Ac-GFIFHIIKGLFHAGRMIHGLVNRRRHRHGMEE-NH2) peptides were synthesized by GL Biochem Ltd. (Shanghai, China), and antimicrobial activity of each peptide was determined as minimum inhibitory concentration (MIC) from a microbroth dilution series [9, 13]. A 102-bp codon-optimized sequence corresponding to the mature EP cDNA gene (g6498.t1) was designed. The sequence was based on the preferential codon usage of *Pichia pastoris* according to the Graphical Codon Usage Analyser (http://gcua.schoedl.de/) and synthesized by Omics Bio (Taipei, Taiwan). The DNA was cloned into EcoRI/XbaI-digested pPICZαA by Omics Bio (Taipei, Taiwan) to create the recombinant vector, pPICZαA-EP-his. The EP cDNA gene (g6498.t1) sequence in the vector was then confirmed by sequencing. pPICZαA-EP-his was linearized by SacI and then transformed into *P*. *pastoris* X-33 by electroporation (1.5 kV, 25 μF, 200 Ω; ECM 399 electroporation system, BTX Harvard Apparatus). Identification of positive transformants and selection of a rEP-expressing clone with high expression was performed as previously reported, without modification [17]. Briefly, the recombinant *P*. *pastoris* strain containing the gene for rEP was incubated in 3 ml YPD medium (with 300 μg Zeocin). After 24 h (30°C), 1 ml *P*. *pastoris* was used to inoculate 30 mL BMGY medium for 24 h (30°C). Cells were then harvested by centrifugation at 6000 ×*g* for 15 min. The cell pellet was resuspended in 50 mL BMMY medium. The supernatant and pellet were examined by SDS–PAGE and Western blot using His-tag antibody (Abcam, ab213204). The recombinant protein was identified by MALDI-TOF (matrix-assisted laser desorption ionization-time of flight) mass spectrometry. The antimicrobial activity of rEP was analyzed using an inhibition zone assay at different treatment temperatures [18]. All assays were performed in triplicate.

**Table 1:** List of primer sequences.

Gene	Forward primer sequence (5'-3')	Reverse primer sequence (5'-3')
g6496.t1^1^	atggcaagtgagcctgacgaac	ttagcctttggtttttcctcctgct
g6497.t1^2^	atgggtgtggcggtgcag	tcatttctgccagtatggcgg
g6498.t1^3^	atgaggtgcatcatcctctttc	tcaggcaaaagctttctctcgttc

^1^g6496.t1: Epinephelus lanceolatus piscidin-1

^2^g6497.t1: Epinephelus lanceolatus piscidin-2

^3^g6498.t1: Epinephelus lanceolatus piscidin-3

### 2.3. Expression of rEP in a fermenter

To optimize the expression conditions in a 5000 ml fermenter, a single rEP colony was used to inoculate 200 ml BMGY with PTM4 medium for 36 h at 28°C, 200 rpm. The culture was then transferred to a 5000 ml fermenter (Winpact, Major Science, Taoyuan, Taiwan) containing 3000 ml commercial fermentation medium (BMGY with PTM4) [19]. During fermentation, the temperature was maintained at 30°C. The pH was adjusted to 6.0 with 14% ammonia and 0.1 N H_2_SO_4_, and dissolved oxygen was maintained above 20% saturation. To produce rEP for supplementation of *G*. *g*. *domesticus* fodder, we expressed rEP in a fermenter using basal salt medium and PTM1 [20]. After glycerol was completely consumed (19 h), 50% w/v glycerol was fed for 360 min. Next, a 100% methanol feed was started and maintained for 24–96 h. The rEP-expressing yeast cultures were then centrifuged at 6000 rpm for 30 min, and supernatants were spray-dried (YC-500, Shanghai Pilotech Instrument & Equipment Co, Ltd.) before mixing into fodder. Before fodder production, rEP expression in each culture was validated by Western blotting, and the rEP content was assessed by comparing with synthetic EP peptide.

### 2.4. Antimicrobial activity of rEP and fodder preparation

The antimicrobial activity of rEP was tested on cultures of *Staphylococcus aureus* (BCRC 10780), *Escherichia coli* (BCRC 10675), *Pseudomonas aeruginosa* (ATCC 19660), and *Riemerella anatipestifer* (RA3, RA9, RA16, CFC27, CFC363, CFC437). The bacteria cultures were derived from single clones that had been expanded and stored at -70°C. Cultures were inoculated in liquid media and cultured overnight at 37°C on a shaker at 180 rpm. Subsequently, the bacteria were diluted in fresh medium (1:1000) and incubated under the same conditions. The four types of bacteria (10^4^ CFU/ml) were mixed with 100 μl of the rEP solution and incubated overnight at 37°C. Supernatant from *P*. *pastoris* transformed with vector alone was used as a control. After 24 h, culture growth was assessed by OD_600_. All assays were performed in triplicate. The composition analysis of diets fed to *G*. *g*. *domesticus* is shown in S1a Table.

### 2.5. *G*. *g*. *domesticus* maintenance and dietary treatment

A total of 189 male or female 2-day-old *G*. *g*. *domesticus* were randomly assigned to seven dietary treatment groups, including rEP (0.75, 1.5, 3.0, 6.0 and 12%), spiraline-A (Juily Pharmaceutical Co., LTD. San Sia, Taipei Hsien, Taiwan), and basal diet groups. Each treatment group consisted of one cage with twenty-seven *G*. *g*. *domesticus*. The composition of the fodder is presented in S1b–S1d Table. The animals were allowed *ad libitum* access to fodder and water for the duration of the trial, beginning from 3 days of age. The total time of feeding was 35 days. The *G*. *g*. *domesticus* were maintained in a cage (animal container) with a constant environment (28.9–37.8°C, 12/12 h light-dark cycle). rEP fodder and fecal sewage were not directly discharged to the outside.

### 2.6. Sample collection and Enzyme-linked immunosorbent assay

Body weight and survival rate were monitored daily during the experimental period. The weight gain, feed efficiency (FE), protein efficiency ratio (PER), and survival percentage were calculated. After 35 days of treatment, the *G*. *g*. *domesticus* were euthanized, blood samples were collected and serum was obtained by centrifugation (3000 ×*g* for 15 min at 4°C). Serum was kept at -80°C until analysis. Enzyme-linked immunosorbent assay (ELISA) was performed in accordance with the manufacturer’s standard procedures to determine the levels of immunological factors, including TNF-α, Interleukin-1β, Interleukin-6, Interleukin-10, Lysozyme, Immunoglobin-G (IgG), and Interferon-γ. Chicken serum samples were analyzed with ELISA kits from ABclonal Inc. (Woburn, MA, USA). After the reaction was completed, the optical density (OD) was measured at 450 nm on a microplate reader (SpectraMax^®^ i3, Molecular Devices, Lagerhausstrasse, Wals, Austria).

### 2.7. Statistical analysis

Data were analyzed with Prism 7 software (GraphPad Inc., La Jolla, CA, USA). Values represent the mean ± standard deviation (SD). *P* < 0.05 was considered significant for one-way Analysis of variance (ANOVA) with Tukey’s multiple comparison test.

## 3. Results

### 3.1. Novel piscidins from *E*. *lanceolatus* exhibit antimicrobial activity

**Fig 1:**
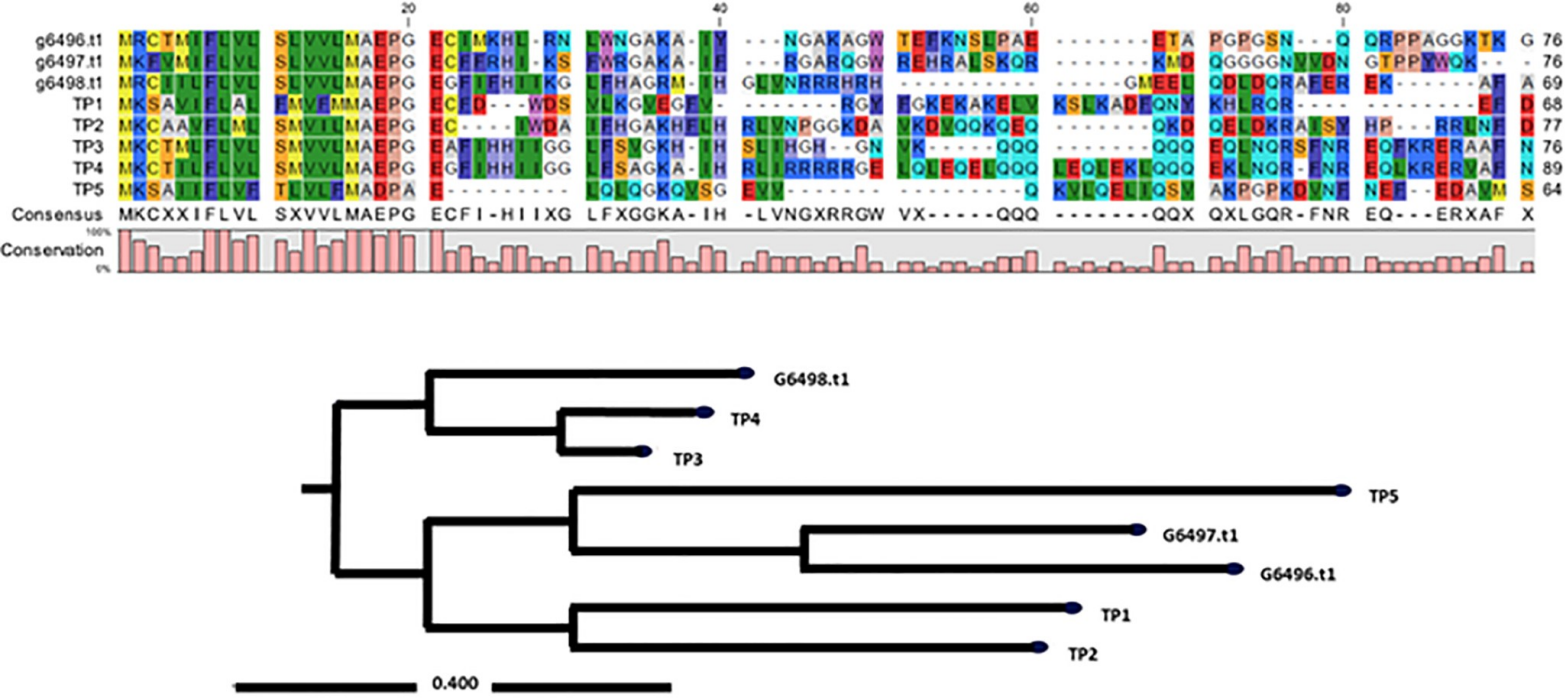
Sequence alignment of tilapia piscidins and *Epinephelus lanceolatus* piscidins. Multiple sequence alignments of the tilapia piscidin peptides with isolated *E*. *lanceolatus* piscidins (g6496.t1, g6497.t1 and g6498.t1). Gaps were inserted to obtain maximum homology. All coding sequences were input into the dendrogram for alignment. The same amino acid is indicated by the same color, e.g., methionine (M) is indicated by yellow. The result of a phylogenetic analysis of piscidins from tilapia (TP1 to TP5) and *E*. *lanceolatus* is shown.

The cDNA coding regions for different piscidin sequences (S1 Fig) were isolated and characterized from *E*. *lanceolatus*. The three identified cDNA sequences were named g6496.t1, g6497.t1 and g6498.t1, and respectively encoded 77, 77 and 70 amino acids. Alignment of *E*. *lanceolatus* piscidins (g6496.t1, g6497.t1, and g6498.t1) and *Oreochromis niloticus* piscidins (TP1-TP5) revealed remarkably high sequence similarities (Fig 1). A phylogenetic tree showed that g6498.t1 corresponded to TP4 and TP3, which exhibit the best antimicrobial activity characterized to date (Fig 1). Next, antimicrobial activity was determined for synthetic g6496.t1, g6497.t1 and g6498.t1 peptides. All three peptides were active against Gram-positive and Gram-negative bacteria, as shown in Table 2. The g6498.t1 and g6497.t1 peptides were also active against Methicillin-resistant *Staphylococcus aureus* (MRSA), with 5.6 μg/ml and 60 μg/ml MICs, respectively. The two peptides were also toxic to *Vibrio parahaemolyticus* at similar MICs. Since g6498.t1 had stronger antimicrobial activity than g6496.t1 and g6497.t1, we named this peptide EP and further produced it in a *P*. *pastoris* protein expression system.

**Table 2:** *In vitro* activity of antibacterial peptides against Gram-positive and Gram–negative bacteria.

Bacteria strain	Gram	G6496-t1^1^	G6497-t1^2^	G6498-t1^3^
*Listeria monocytogenes*	+	ND	ND	ND
*Staphylococcus aureus subsp*	+	60	60	90
*Streptococcus equinus*	+	30	15	11.2
*MRSA*	+	ND	60	5.6
*Streptococcus agalactiae*	+	60	60	90
*Escherichia coli*	_	60	30	22.5
*Vibrio parahaemolyticus*	_	ND	60	5.6
*Vibrio alginolyticus*	_	ND	ND	ND
*Klebsiella oxytoca*	_	60	30	45
*Pseudomonas aeruginosa*	_	ND	ND	45

^1^g6496.t1: Epinephelus lanceolatus piscidin-1

^2^g6497.t1: Epinephelus lanceolatus piscidin-2

^3^g6498.t1: Epinephelus lanceolatus piscidin-3

(the MIC units are in μg/ml)

### 3.2. *P*. *pastoris* expression system for rEP peptide

**Fig 2:**
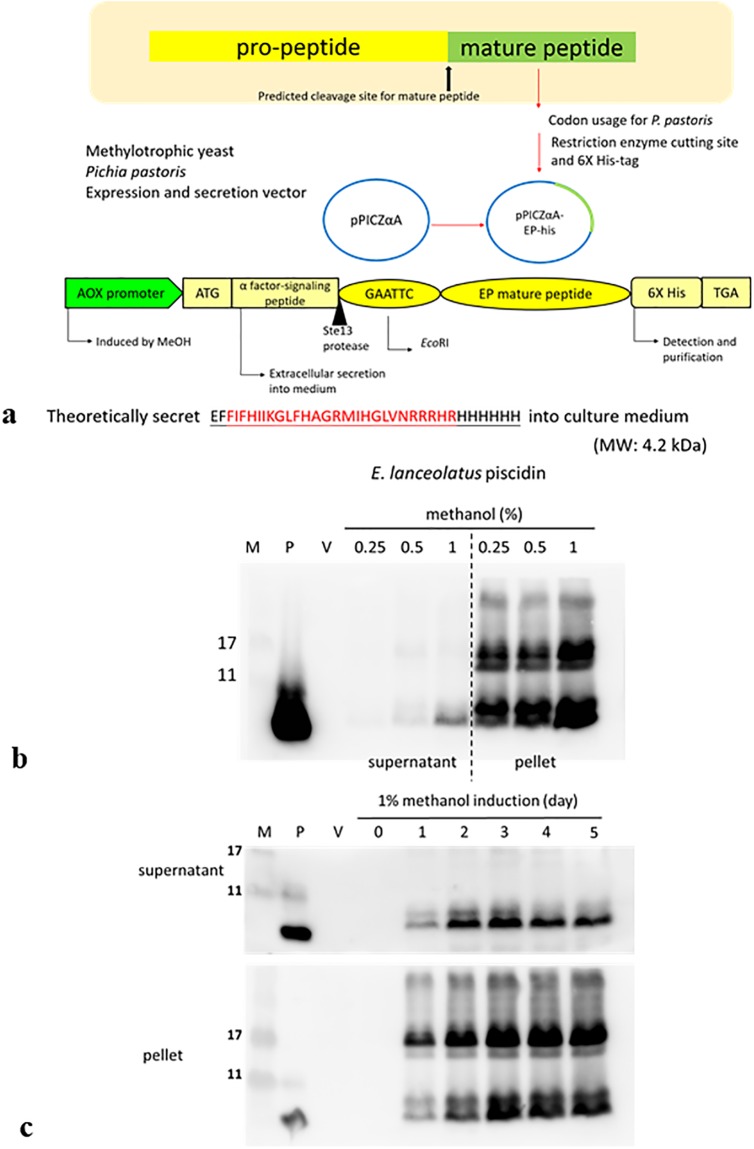
Expression of the *E*. *lanceolatus* piscidin-6×His (rEP) protein in *Pichia pastoris*. (a) Plasmid map of the pPICZαA-EP-his vector. (b) Different concentrations of methanol were used for induction, and recombinant protein expression was analyzed by western blotting. (c) Cells were harvested and total protein from supernatant and pellet were analyzed by SDS-PAGE and western blotting. Lane 1, low-range rainbow marker; lane 2, synthesized EP; lane 3, protein expressed by the pPICZαA vector; lane 4–9, cells containing EP expression vector after induction for 0 h (no methanol induction), 1, 2, 3, 4 and 5 days.

As shown in Fig 2a, the constitutive expression vector pPICZαA-EP-his contains a methanol-inducible *AOX* promoter, an α-factor signaling peptide, and the *STE13* gene for Dipeptidyl aminopeptidase A. The amino acid sequence for rEP is encoded by a gene insert that is codon-optimized for *P*. *pastoris*. Transformants (*P*. *pastoris* X-33) were grown on a Zeocin plate (25 μg/ml) and screened by colony hybridization with anti-His antibody; clones with high expression were selected for subsequent experiments. After identification of clones, we validated the presence of a correct expression cassette by PCR amplification and DNA sequencing. During the initial expression experiments, the optimal methanol concentration was determined by supplying different concentrations of methanol for 24 h. The supernatants and pellet (after centrifugation) were collected and analyzed by Western blotting. The results showed that 1% methanol induced robust expression of rEP (Fig 2b). The time-dependent effects of 1% methanol induction are shown in Fig 2c. Thus, 1% methanol induction was selected for subsequent fermenter experiments.

### 3.3. Effects of time and medium on fermenter production of rEP

**Fig 3:**
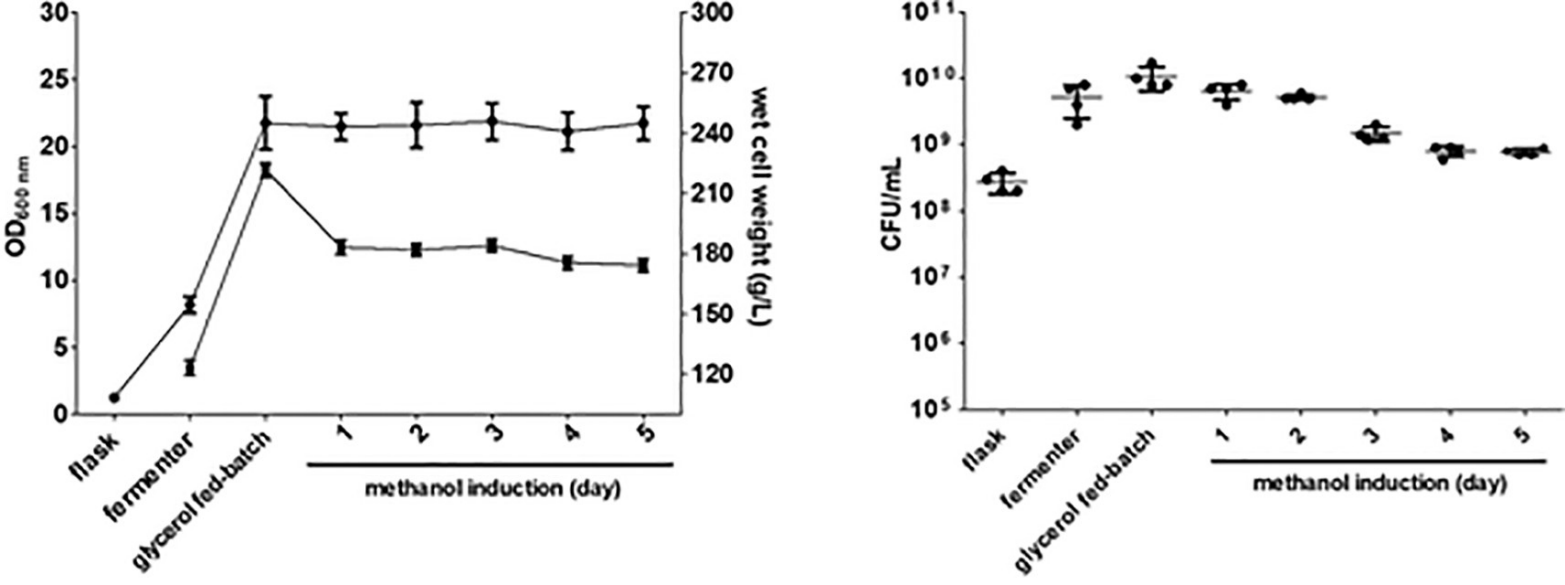
Effect of medium composition on the expression of rEP in *P*. *pastoris*. The effect of nutrient content was evaluated by comparing *P*. *pastoris* X33 transformant cultures grown in BMGY (flask culture, circles) and BSM (fermenter culture, squares) according to thier wet cell weight and cell counts.

**Fig 4:**
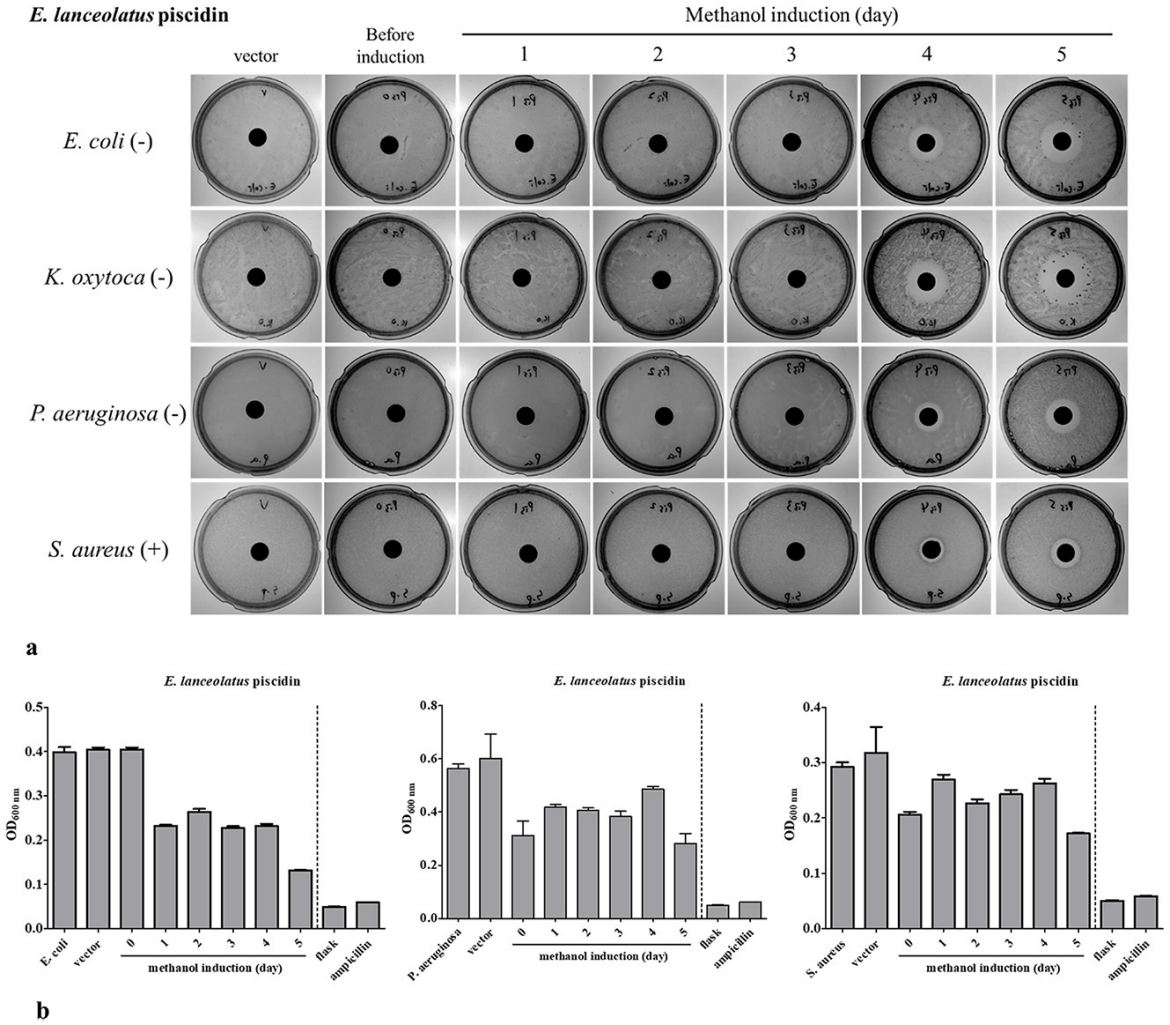
Antimicrobial activity of rEP produced in *P*. *pastoris* by flask and fermenter methods. (a) rEP concentration in the yeast culture supernatant before and after induction with methanol for 24 h to 120 h in flask culture. After 5 days of induction, yeast culture supernatant produced large inhibition zones, ordered by width, for *K*. *oxytoca*, *E*. *coli*, *P*. *aeruginosa*, and *S*. *aureus*. The marker “-” represents Gram-negative; “+” represents Gram-positive. (b) The antibacterial activity of rEP produced by the fermenter method. Yeast were induced with methanol from 24 h to 120 h in a fermenter. The supernatant showed inhibitory activity toward *E*. *coli*, *P*. *aeruginosa*, and *S*. *aureus*, as measured by OD_600_. The flask supernatant exhibited greater antimicrobial activity compared to fermenter supernatant.

To evaluate the effects of induction time and medium on rEP expression, transformants were grown in a fermenter with basal salt medium (BSM) or BMGY. Large-scale production of rEP from *P*. *pastoris* (X-33) may be most efficient with high-density cultivation methods. Thus, rEP induction was evaluated in a 5000 mL fermenter. When the wet weight of cells reached 200 g/L (16–18 h post-inoculation) in a glycerol-fed batch, 1% methanol was added for times ranging from 24 to 120 h. After methanol induction, the yeast number decreased (Fig 3). The total rEP protein levels reached maxima of 0.8 mg/L in supernatant and 5.63 mg/L in pellet after 48 h induction in BSM medium. On the other hand, the total maximum rEP protein levels in BMGY medium were 3.6 mg/L in supernatant and 7.74 mg/L in pellet at 48 h. The antimicrobial activity of rEP was also monitored, gradually increasing to its highest level after 120 h methanol induction (Fig 4a and 4b, S2 Fig).

### 3.4. Antimicrobial activity of EP

The antimicrobial activity of rEP from flask cultures was evaluated by determining its ability to prevent growth of Gram-positive and Gram-negative bacteria with a disk diffusion assay. Table 3 shows that rEP exhibited broad-spectrum antimicrobial activity against the tested bacterial strains. The most susceptible pathogens were *R*. *anatipestifer* (CFC27) and *R*. *anatipestifer* (CFC437) (Table 3a). Next, we investigated the thermostability of flask-fermented rEP with regard to its toxicity toward *S*. *aureus* (BCRC 10780), *E*. *coli* (BCRC 10675) and *P*. *aeruginosa* (ATCC 19660). Empty vector with no insert in *P*. *pastoris* was used as a control. Thermostability was measured by disk diffusion assay after cultures were incubated for 5 min at 40, 60, 80, or 100°C (Table 3b). We found that the inhibition zone diameter (mm) values were decreased by increasing pretreatment temperature, but the antimicrobial activity was not affected by different temperatures in *Staphylococcus aureus* (BCRC 10780). This finding suggests that the tertiary (or secondary) structure of rEP is important for peptide stability, and it should be protected from high temperatures to maintain activity.

**Table 3:** The antimicrobial activity and effect of temperature of rEP. The rEP was produced in *P*. *pastoris* using a shake flask. Supernatant was collected 120 h after induction and applied to disk paper at the maximum carrying capacity. The disk paper was placed on a bacterial culture plate for 16 h at 37°C. Inhibition zone diameters were measured. Representative radial diffusion assays are shown. (a) The antimicrobial activity of rEP. (b) The effect of temperature on rEP antimicrobial activity.

a			
Microorganism	Inhibition zone diameter (mm)		
	Vector control^2^	rEP	Ampicillin (2 mg/ml)
*Escherichia coli* (BCRC 10675)	NI^1^	8.6±0.5	13.6±0.5
*Pseudomonas aeruginosa* (ATCC 19660)	NI	10.0±1.0	11.6±0.5
*Staphylococcus aureus* (BCRC 10780)	NI	10.6±1.1	14.6±0.5
*Riemerella anatipestifer* (RA9)	NI	NI	31±0.8
*Riemerella anatipestifer* (CFC27)	NI	12, 14	43±1.1
*Riemerella anatipestifer* (CFC437)	NI	11, 13	15±4.5
*Riemerella anatipestifer* (RA3)	NI	10, 10	12±2.5
*Riemerella anatipestifer* (CFC363)	NI	NI	32±2.3
*Riemerella anatipestifer* (RA16)	NI	NI	30±1.2

b				
Temperature	Inhibition zone diameter (mm)			
	Vector control^2^	*Staphylococcus aureus* (BCRC 10780)	*Pseudomonas aeruginosa* (ATCC 19660)	*Escherichia coli* (BCRC 10675)
40°C	NI^1^	10.6±1.5	10.1±0.3	9.0±1.0
60°C	NI	9.1±0.7	9.5±0.2	9.0±1.0
80°C	NI	9.3±0.5	NI	8.6±0.5
100°C	NI	9.3±0.5	NI	NI

^1^NI, no inhibition.

^2^ Vector control was fermentation supernatant from transformants harboring empty plasmid. Ampicillin (2 mg/ml) was applies at a volume of 10 μl to disk paper.

### 3.5. rEP supplementation improves growth performance and immune response

**Fig 5:**
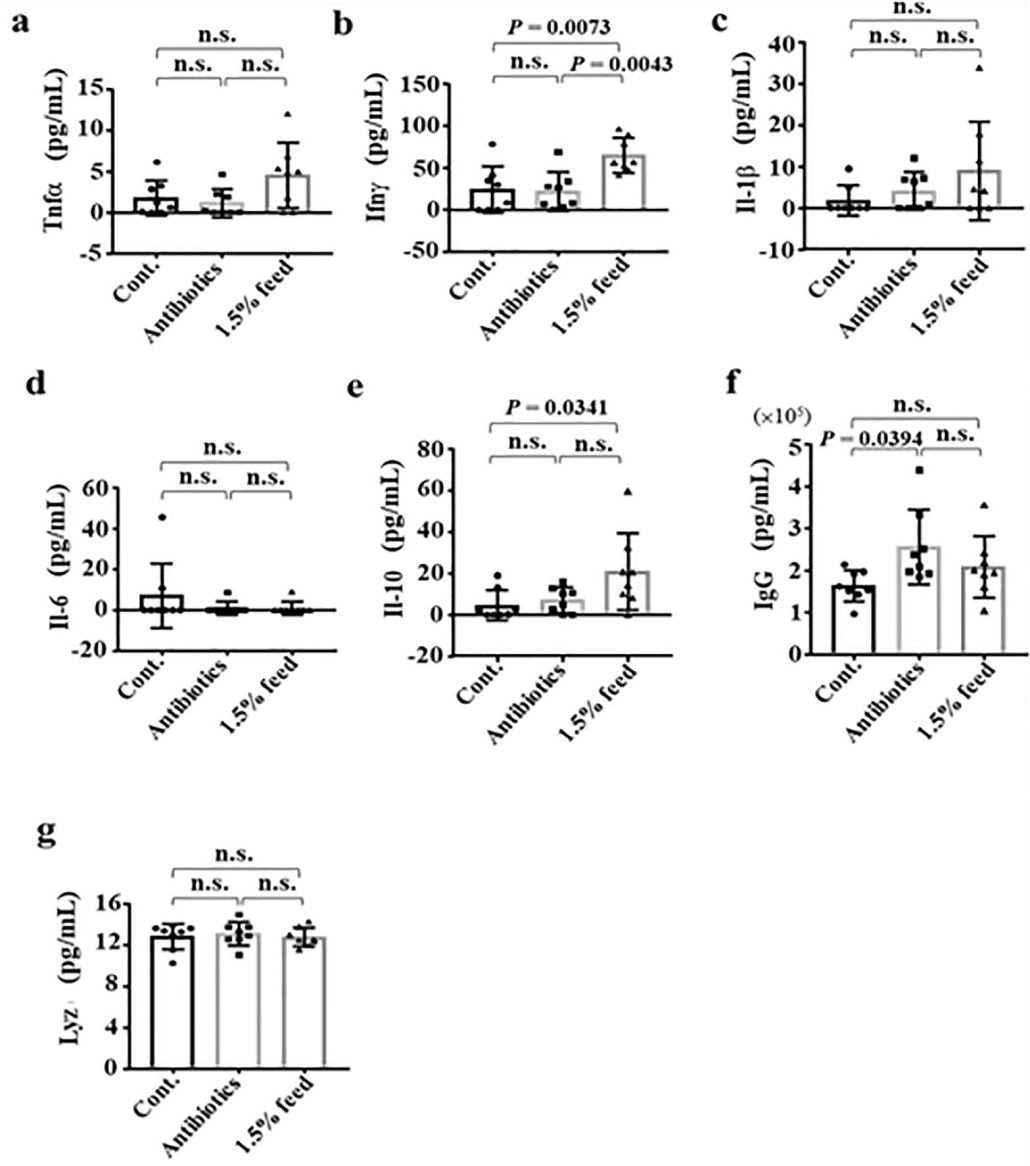
Effects of rEP administration in chicken were evaluated by determining the levels of immunological factors, including immunoglobin G (IgG), Tumor necrosis factor (TNF)-α, Interleukin (IL)-1β, IL-6, IL-10, Lysozyme (Lyz), and Interferon (IFN)-γ by ELISA.

The growth performance of *G*. *g*. *domesticus* was evaluated in terms of weight gain and feed efficiency (FE). At the end of the 35-day experimental period, animals fed with 1.5% and 3.0% rEP had better growth performance than those in the spiraline-A and basal diet groups (Table 4). The physiological effects of daily rEP administration in chicken were then evaluated by determining the levels of immunological factors in the serum, including immunoglobin G (IgG), Tumor necrosis factor (TNF)-α, Interleukin (IL)-1β, IL-6, IL-10, Lysozyme (Lyz) and Interferon (IFN)-γ by ELISA (Fig 5a and 5g). No significant changes between groups were observed for TNF-α, Il-1β, Il-6 or Lyz levels (Fig 5a, 5c, 5d, and 5g). The group supplemented with 1.5% EP showed significantly increased IFN-γ level compared to the antibiotics and control groups (Fig 5b, *P* = 0.0043 compared to antibiotic group, and *P* = 0.0073 compared to the control group). In addition, the IL-10 level was significantly higher in the rEP-supplemented group than in the control group (*P* = 0.0341) but was not different than the antibiotic group (Fig 5e). Chicken fed with antibiotics showed significantly higher serum IgG levels compared to the control group (*P* = 0.0394) (Fig 5f).

**Table 4:** Weight gain, feed efficiency (FE), protein efficiency ratio (PER) and survival of *Gallus gallus domesticus* fed with diets containing rEP (0.75, 1.5, 3.0, 6.0 and 12%) of fermentation supernatant spray-dried powder for 4 weeks^1^.

Group	Weight gain ^2^ (%)	FE ^3^	PER ^4^ (%)	Survival (%)
Spiraline-A (Commercially available)	2669.29±168.79	0.56±0.03	1.74±0.0779	100%
Basal diet	2356.26±116.74	0.52±0.03	1.54±0.08	100%
0.75% rEP	2531.80±142.13	0.53±0.03	1.58±0.09	92.60%
1.5% rEP	2727.42±116.08	0.58±0.03	1.8±0.08	100%
3.0% rEP	2809.71±119.30	0.57±0.02	1.86±0.08	96.20%
6.0% rEP	2738.11±109.04	0.57±0.02	2.03±0.08	100%
12.0% rEP	2181.26±158.95	0.48±0.04	2.17±0.16	96.20%

^1^ Values in the same column with different superscript are significantly different (p<0.05). Data are expressed as mean ± SD from group of chicken (n = 27).

^2^ Weight gain (%) = {Final body weight (g)–Initial body weight (g)}/ Initial body weight (g) x 100

^3^ Feed efficiency = {Final body weight (g)–Initial body weight (g)}/ Feed intake (g)

^4^ Protein efficiency ratios = {Final body weight (g)–Initial body weight (g)}/ Protein intake (g)

## 4. Discussion

For many years, antibiotics have been added to chicken fodder to prevent pathogen infection. However, research has suggested that the overuse of antibiotics may lead to resistance in pathogens. Antibiotics remaining in fouled feed or those released into the environment may be responsible for creating multiple antibiotic-resistant bacteria, potentially giving rise to a global public health crisis [21, 22]. Thus, substitutes for antibiotics are in high demand for livestock breeding. One class of new drug candidates considered for this purpose is AMPs, which have excellent antimicrobial activity toward pathogens [23]. Notably, our previous studies have shown that in addition to antimicrobial activity, fish piscidins also possess anti-cancer activity [9, 11, 13]. In order to study the effects of AMPs as fodder supplements in agriculture, large amounts of active peptide must be produced. In this study we accomplished this goal using a yeast expression system.

Three cDNAs encoding putative antimicrobial piscidin peptides were isolated from *E*. *lanceolatus* and characterized in this study. The transcripts were named g6496.t1, g6497.t1 and g6498.t1, and respectively encode putative AMPs of 77, 77, and 70 amino acid residues. Based on sequence alignments between tilapia piscidins and g6496.t1, g6497.t1 and g6498.t1, we found that g6498.t1 (named EP) exhibits high similarity to the highly active TP3 and TP4 peptides from tilapia. Although piscidins are widely distributed in skin mucus, spleen, and blood of many teleost species [24], we chose to perform alignments only with tilapia piscidins, which are among the best characterized AMPs. Antimicrobial activities were examined by determining the MICs, allowing us to evaluate the strength of antimicrobial activity and choose a single piscidin gene for protein expression. The experimental results suggested that g6498.t1/EP peptide had better activity than g6496.t1 or g6497.t1 and were consistent with previous reports that piscidin family members possess antimicrobial or growth inhibition activity in Gram-negative and Gram-positive bacteria [24, 25].

Recently, piscidins were expressed and purified in *E*. *coli* for an NMR study [26]. Another study reported that recombinant piscidin protein expression could be achieved in *E*. *coli* at around 15 mg/L in Luria broth or 1.5 mg/L in minimal medium [15]. However, neither paper assessed antimicrobial activity of the recombinant piscidin peptide. In this study, we expressed rEP using a gene with optimized yeast codons in the pPICZαA expression vector for *P*. *pastoris* [27]. Since EP is a fish gene, we expected that optimization of the gene for *Pichia* codon usage would increase yield. Notably, other researchers have reported difficulties in producing high levels of AMPs due to degradation by host proteolytic enzymes [15]. In our study, we did not find that proteolysis prevented substantial accumulation of rEP in *Pichia*.

His-tagged rEP expressed in *P*. *pastoris* showed antimicrobial activity against Gram-positive and Gram-negative bacteria. Several other AMPs, such as tachyplesin [28], defensin [29], and human neutrophil peptide 1 (HNP1) [30], have also been successfully expressed in *P*. *pastoris* and showed strong antibacterial activities. It was previously reported that the antimicrobial activities of tilapia piscidins are highly stable over a temperature range from 40 to 100°C and a pH range from 4.0 to 12.0 [31]. Similarly, piscidins from *E*. *lanceolatus* that were expressed in *P*. *pastoris* were found to be relatively stable *in vitro* after incubation in extreme temperature conditions (100°C), still retaining some antimicrobial activity against *S*. *aureus* (BCRC 10780). The stability of rEP may be related to its high arginine content. In line with this notion, it was previously reported that increasing arginine composition of peptides increases antimicrobial activity and enhances the ability of peptides to embed into membranes [32]. Increased arginine content may also enhance the translocation and membrane permeabilization functions [33]. A previous report showed the activity of another antimicrobial peptide (recombinant protegrin-1) remained stable over a broad temperature range (25°C to 95°C), possibly due to its high cysteine content (disulfide bonds) [34]. However, rEP may change conformation after treatment with high temperatures of 80°C or 100°C. Such conformation changes may be responsible for the decreased antimicrobial activity of rEP against Gram-negative bacteria, while leaving activity toward Gram-positive bacteria unaffected. Notably, rEP should be able to adopt different conformations depending on the surrounding microenvironment and experimental conditions. Moreover, Gram-negative bacteria contain moderate amounts of anionic lipids (below 20%) in their outer and cytoplasmic membranes, whereas the cytoplasmic membrane of Gram-positive bacteria is rich in anionic lipids (more than 50%) [35]. We saw that the antimicrobial activity of rEP was proportional to its helical structure, irrespective of its composition and overall negative charge. Our experimental results thus conform to the prevailing model that electrostatic attraction of antimicrobial peptides to anionic membranes is the first thermodynamic step in antimicrobial peptide action; this step can energetically compensate for heat release due to the formation of α-helices and β-sheets [36].

In this study, we show that fodder supplementation with crude rEP improved the growth performance of *G*. *g*. *domesticus* compared to Spiraline-A-supplemented fodder. These results suggest that EP can potentially replace antibiotics to improve growth performance. Since the thermostability of rEP is questionable, we can propose two solutions for its application. First, rEP could mixed into fodder just prior to feeding chickens, avoiding a heating step. Second, since a heating process is usually incorporated in commercial fodder preparation, the thermostability of rEP dietary supplement may be improved by modifying the structure before further development of the product for animal husbandry. Nevertheless, rEP showed positive effects using the protocols in this study.

The growth promoting action of EP may be related to intestinal absorption and as such, small villus morphology was evaluated in a previous study. Histological analysis showed that duodenum villus height was significantly greater in rEP animals than that in the control group (S3a–S3d Fig). Moreover, growth enhancement has been observed in other reports [37, 38]. For example, feeding mice with PR39 peptide (4.7 kDa proline-rich AMP)-expressing *Lactobacillus casei* caused jejunum and duodenum villus height in the intestinal villi to increase [39]. Use of recombinant porcine β-defensin 2 (rpBD2) as a feed additive for weaned piglets also produced increases in intestinal villus height of the duodenum and jejunum [40]. Additionally, birds fed with AMP-A3 diet had increased villus height of the duodenum, jejunum and ileum [41]. Several articles have suggested that the beneficial effects of oral AMP administration on animal gut morphology could be due to reductions in the population of harmful bacteria [41, 42]. In our study, we found that 1.5% rEP significantly increased the numbers of Lactobacillaceae and Entercoccaceae, while decreasing the numbers of Enterobacteriaceae and Staphylococcaceae in duodenum when compared to spiraline A and basal diet groups (S3e Fig). Other studies have shown that administration of AMPs can also significantly decrease pathogens, such as *Salmonella*, in mice and turkeys [42, 43].

After daily rEP administration, IL-10 was significantly higher in the rEP-supplemented group compared to the control group. IL-10 is a proinflammatory cytokine, which is produced by different cells, such as Th2 cells, macrophages and monocytes, and functions as an immunoregulator during infection with bacteria, fungi and viruses. The higher IFN-γ production in rEP-supplemented animals may increase cytokines that induce antimicrobial pathways to protect against extracellular and intracellular pathogens [44].

## Conclusions

The current study describes the beneficial use of a novel fish AMP expressed by *P*. *pastoris* as a supplement that may replace antibiotics in *G*. *g*. *domesticus* feed. The results indicate that supplementation of 1.5% or 3% rEP in chicken fodder can improve growth performance, intestinal morphology, microbiota and immunity in broilers. Further research will be needed to study the effects of different dosages, evaluate potential toxicity, determine the effectiveness at mitigating *Salomnella* infection, and confirm the benefits of dietary supplementation. These studies should be performed in large numbers of *G*. *g*. *domesticus* within a chicken farm setting. Our research results further suggest there may be a substantial economic benefit to using genetically modified organism additives, such as piscidin-expressing yeast, for delivery of fish recombinant AMPs in poultry feeds.

## Supporting information

S1 Fig Sequences of *E*. *lanceolatus* piscidins g6496.t1, g6497.t1 and g6498.t1. Nucleotide (nt) sequences and predicted amino acid (aa) sequences are shown. Nucleotides are numbered beginning with the first nucleotide. Asterisk (*) indicates a stop codon. The EP cDNA gene (g6498.t1) was modified based on the preferential codon usage of *P*. *pastoris* expression system.
(TIF)

S2 Fig The effects of recombinant *Epinephelus lanceolatus* piscidin (EP) and bacterial culture co-incubation against Gram-positive and Gram-negative bacteria. Use OD600 nm as measured unit. Lower OD600 represent the bacteria was inhibited growth by recombinant *Epinephelus lanceolatus* piscidin (EP). Vector control is mean protein expressed by the pPICZαA vector alone. EP is mean protein expressed by the pPICZαA-EP vector.
(TIF)

S3 Fig Effects of orally administered a rEP on posture, body weight, intestinal morphology, and gut flora. (a) After 28 days of feeding the 1.5% rEP-receiving chickens were larger than the spiraline A and basal diet controls. (b) The effects of feeding 1.5% rEP on body weight. (c) Representative picture of hematoxylin and eosin staining of intestinal villi and crypts. (d) The intestinal villus length and crypt depth were measured. (e) The effects of orally administrated the spiraline A, basal diet, and 1.5% rEP on intestinal microflora in *G*. *g*. *domesticus*. Relative proportions of bacterial families are shown. Total viable counts of Enterobacteria and Staphylococca were reduced, while the abundance of Lactobacillaceae and Enterococcaceae were increased in the duodenum.
(TIF)

S1 Table Formulation of the basal diet. Table 1a. Proximate analysis of basal feed with additive and commercial feed composition. Table 1b. Formulation of the basal diet of early stage. Table 1c. Formulation of the basal diet of middle stage. Table 1d. Formulation of the basal diet of late stage.
(DOC)

S1 Raw images (PDF)
