# Supplementary figures and images for: Dietary supplementation of recombinant antimicrobial peptide Epinephelus lanceolatus piscidin improves growth performance and immune response in Gallus gallus domesticus
Source: PLoS One. 2020 Mar 11;15(3):e0230021. doi: 10.1371/journal.pone.0230021 (PMC7065771; doi:10.1371/journal.pone.0230021)

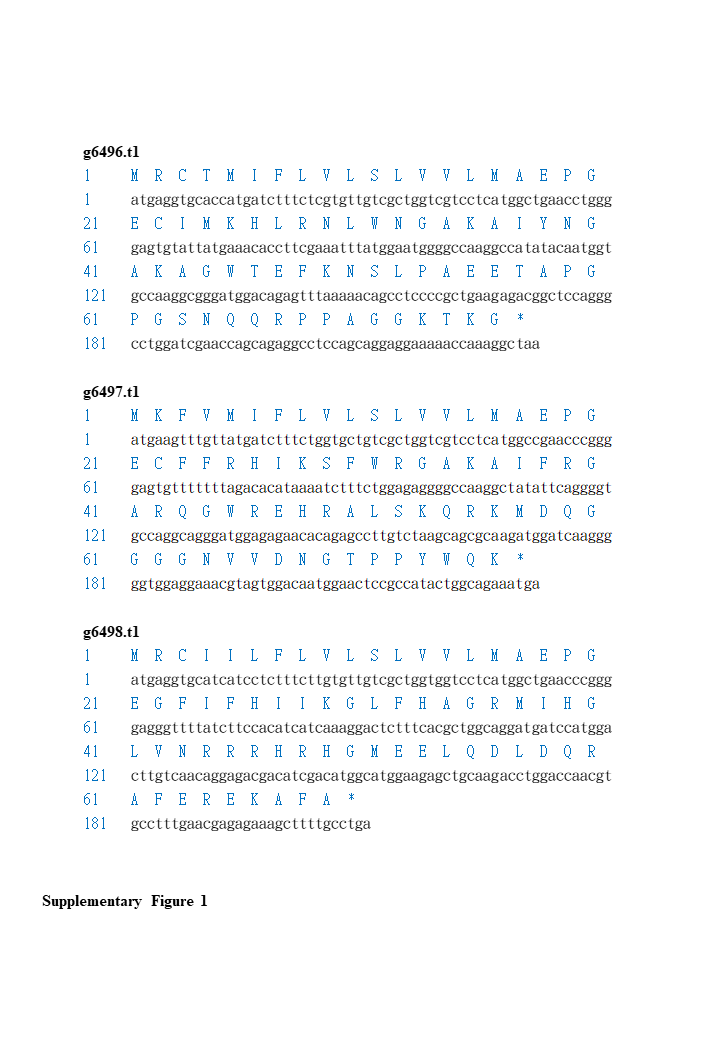

Supplement: S1 Fig — Nucleotide (nt) sequences and predicted amino acid (aa) sequences are shown. Nucleotides are numbered beginning with the first nucleotide. Asterisk (*) indicates a stop codon. The EP cDNA gene (g6498.t1) was modified based on the preferential codon usage of P. pastoris expression system. (TIF) [file pone.0230021.s001.TIF]

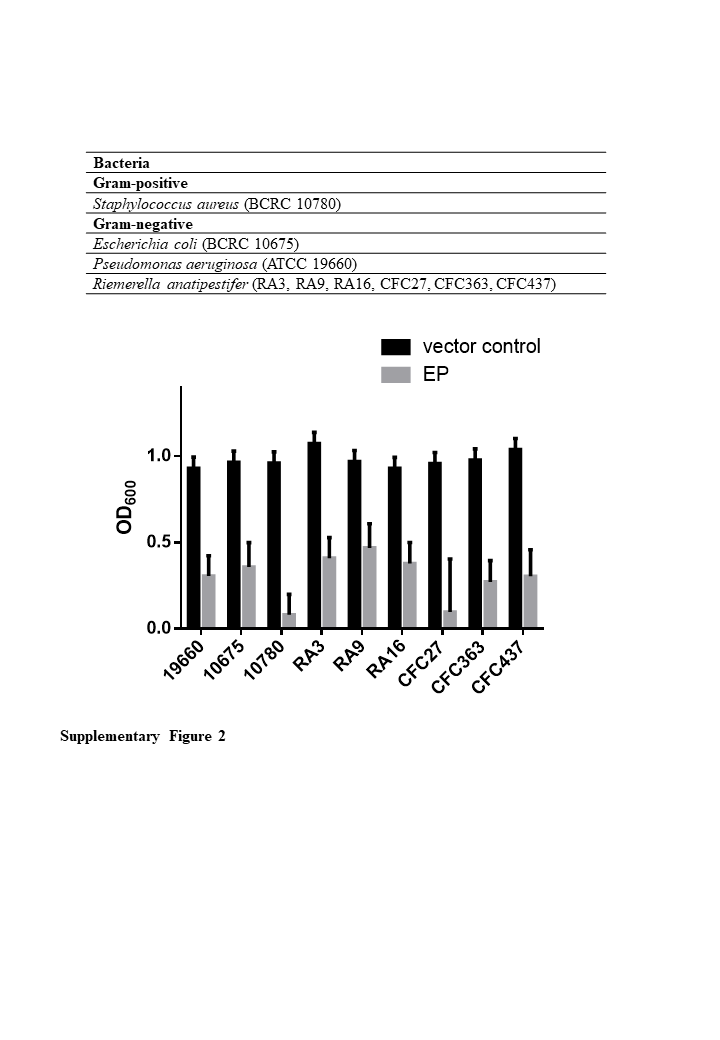

Supplement: S2 Fig — Use OD600 nm as measured unit. Lower OD600 represent the bacteria was inhibited growth by recombinant Epinephelus lanceolatus piscidin (EP). Vector control is mean protein expressed by the pPICZαA vector alone. EP is mean protein expressed by the pPICZαA-EP vector. (TIF) [file pone.0230021.s002.TIF]

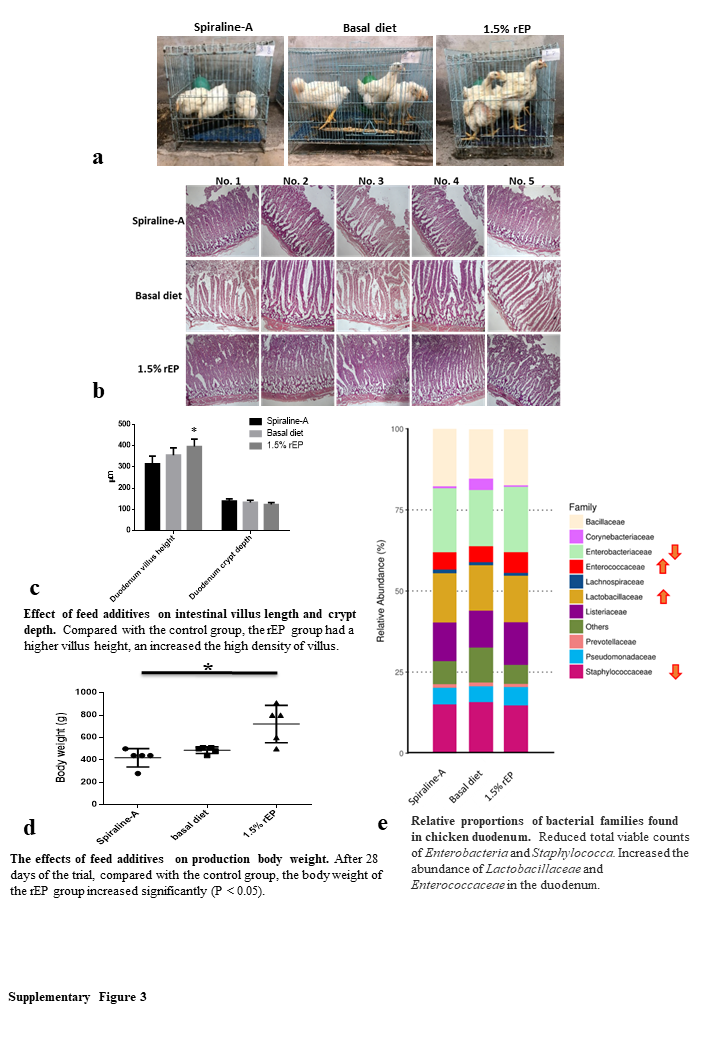

Supplement: S3 Fig — (a) After 28 days of feeding the 1.5% rEP-receiving chickens were larger than the spiraline A and basal diet controls. (b) The effects of feeding 1.5% rEP on body weight. (c) Representative picture of hematoxylin and eosin staining of intestinal villi and crypts. (d) The intestinal villus length and crypt depth were measured. (e) The effects of orally administrated the spiraline A, basal diet, and 1.5% rEP on intestinal microflora in G. g. domesticus. Relative proportions of bacterial families are shown. Total viable counts of Enterobacteria and Staphylococca were reduced, while the abundance of Lactobacillaceae and Enterococcaceae were increased in the duodenum. (TIF) [file pone.0230021.s003.TIF]
