# Supplementary material for: Dietary supplementation of recombinant antimicrobial peptide Epinephelus lanceolatus piscidin improves growth performance and immune response in Gallus gallus domesticus
Source: PLoS One. 2020 Mar 11;15(3):e0230021. doi: 10.1371/journal.pone.0230021 (PMC7065771; doi:10.1371/journal.pone.0230021)
Supplement: S1 Table — Table 1a. Proximate analysis of basal feed with additive and commercial feed composition. Table 1b. Formulation of the basal diet of early stage. Table 1c. Formulation of the basal diet of middle stage. Table 1d. Formulation of the basal diet of late stage. (DOC) [file pone.0230021.s004.doc]

**Supplementary Table 1** Formulation of the basal diet

Table 1a. Proximate analysis of basal feed with additive and commercial feed composition

| Composition |  | | |  | |  |
| --- | --- | --- | --- | --- | --- | --- |
| Basal feed with additive | | Early stage | Middle stage | | Last stage | |
| Crude protein | | 22.54±1.67 |  |  | | --- | --- | --- | | | | 20.99±0.54 | | 20.22±1.32 |
| Crude lipid | | 6.69±0.75 |  |  | | --- | --- | --- | | | | 6.75±0.69 | | 4.77±1.23 |
| Ash | 7.14±1.01 | | | 7.67±1.23 | | 7.19±1.43 |
| Commercial feed | | Early stage | Middle stage | | Last stage | |
| Crude protein | ≦18% | | | ≦15% | | ≦12% |
| Crude lipid | ≦2.5% | | | ≦2.5% | | ≦2.5% |
| Ash | ≧9% | | | ≧10% | | ≧10% |

Table 1b. Formulation of the basal diet ^1^ of early stage.

| Ingredients (%) | Basal diet | 0.75% rEP | 1.5% rEP | 3% rEP | 6% rEP | 12% rEP | 0.2% Antibiotic^2^ |
| --- | --- | --- | --- | --- | --- | --- | --- |
| Yellow corn | 54.3 | 54.3 | 54.3 | 54.3 | 54.3 | 54.3 | 54.3 |
| Soybean meal | 38 | 37.25 | 36.5 | 35 | 32 | 26 | 37.8 |
| Dicalcium phosphate | 1.4 | 1.4 | 1.4 | 1.4 | 1.4 | 1.4 | 1.4 |
| Calcium carbonate | 1 | 1 | 1 | 1 | 1 | 1 | 1 |
| Iodized salt | 0.3 | 0.3 | 0.3 | 0.3 | 0.3 | 0.3 | 0.3 |
| Soybean oil | 3 | 3 | 3 | 3 | 3 | 3 | 3 |
| Vitamin premix | 2 | 2 | 2 | 2 | 2 | 2 | 2 |
| Additive | 0 | 0.75 | 1.5 | 3 | 6 | 12 | 0.2 |

Table 1c. Formulation of the basal diet ^1^ of middle stage.

| Ingredients (%) | Basal diet | 0.75% rEP | 1.5% rEP | 3% rEP | 6% rEP | 12% rEP | 0.2% Antibiotic^2^ |
| --- | --- | --- | --- | --- | --- | --- | --- |
| Yellow corn | 58.2 | 58.2 | 58.2 | 58.2 | 58.2 | 58.2 | 58.2 |
| Soybean meal | 34 | 33.25 | 32.5 | 31 | 28 | 22 | 33.8 |
| Dicalcium phosphate | 1.4 | 1.4 | 1.4 | 1.4 | 1.4 | 1.4 | 1.4 |
| Calcium carbonate | 1.1 | 1.1 | 1.1 | 1.1 | 1.1 | 1.1 | 1.1 |
| Iodized salt | 0.3 | 0.3 | 0.3 | 0.3 | 0.3 | 0.3 | 0.3 |
| Soybean oil | 3 | 3 | 3 | 3 | 3 | 3 | 3 |
| Vitamin premix | 2 | 2 | 2 | 2 | 2 | 2 | 2 |
| Additive | 0 | 0.75 | 1.5 | 3 | 6 | 12 | 0.2 |

Table 1d. Formulation of the basal diet ^1^ of late stage.

| Ingredients (%) | Basal diet | 0.75% rEP | 1.5% rEP | 3% rEP | 6% rEP | 12% rEP | 0.2% Antibiotic^2^ |
| --- | --- | --- | --- | --- | --- | --- | --- |
| Yellow corn | 60.2 | 60.2 | 60.2 | 60.2 | 60.2 | 60.2 | 60.2 |
| Soybean meal | 29 | 28.25 | 27.5 | 26 | 23 | 17 | 28.8 |
| Dicalcium phosphate | 1.3 | 1.3 | 1.3 | 1.3 | 1.3 | 1.3 | 1.3 |
| Wheat bran | 3 | 3 | 3 | 3 | 3 | 3 | 3 |
| Calcium carbonate | 1.2 | 1.2 | 1.2 | 1.2 | 1.2 | 1.2 | 1.2 |
| Iodized salt | 0.3 | 0.3 | 0.3 | 0.3 | 0.3 | 0.3 | 0.3 |
| Soybean oil | 3 | 3 | 3 | 3 | 3 | 3 | 3 |
| Vitamin premix | 2 | 2 | 2 | 2 | 2 | 2 | 2 |
| Additive | 0 | 0.75 | 1.5 | 3 | 6 | 12 | 0.2 |

^1^ Fermentation supernatant spray dry powder was added to diets at the expense of cellulose to provide concentration of 0, 0.75, 1.5, 3.0, 6.0 and 12 g/100 g diet.

^2^ Antibiotic: each gm contains: Spiramycin Adipate 30mg(pot.) Streptomycin Sulfate 30mg(pot.) Vitamin A 2,500I.U. Vitamin B1 5mg Vitamin B2 10mg Vitamin B6 2mg Vitamin B12 5mcg Vitamin E 2mg Vitamin D3 500I.U. Vitamin K4 1mg Folic Acid 0.2mg Calcium Pantothenic Acid 5mg Nicotinic Acid 10mg Lysine 20mg
