# Supplementary material for: Dietary supplementation of recombinant antimicrobial peptide Epinephelus lanceolatus piscidin improves growth performance and immune response in Gallus gallus domesticus
Source: PLoS One. 2020 Mar 11;15(3):e0230021. doi: 10.1371/journal.pone.0230021 (PMC7065771; doi:10.1371/journal.pone.0230021)
Supplement: S1 Raw images — (PDF) [file pone.0230021.s005.pdf]

For Western blot, membranes were incubated in blocking buffer (0.1 M PBS, 5% non-fat milk, 0.1% Tween-20) for 1 h at room temperature and then incubated in the same solution with 6×His antibody (1:5,000, ThermoFisher Scientific) and donkey anti-mouse secondary antibody (GE Healthcare Life Science). Signal was visualized with enhanced chemiluminescence (Immobilon Western Chemiluminescent HRP substrate, Merck Millipore) and detected by an imaging system (UVP, BioSpectrumTM 500).

Expression of the *E. lanceolatus* piscidin-6×His (rEP) protein in *Pichia pastoris*. Different concentrations of methanol were used for induction, and recombinant protein expression was analyzed by western blotting.

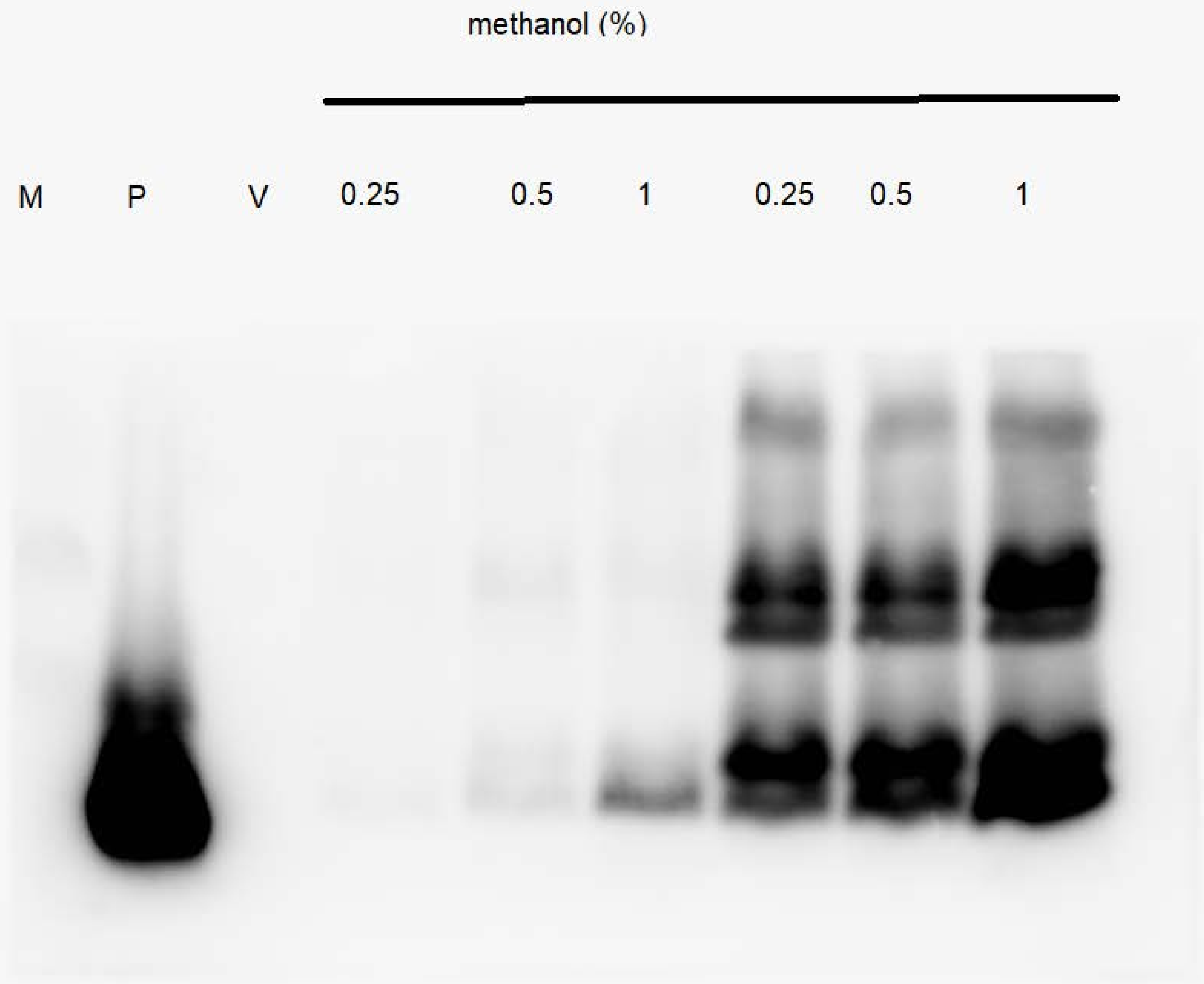

Figure 2b was generated from that original image

Cells were harvested and total protein from supernatant and pellet were analyzed by SDS-PAGE and western blotting. Lane 1, low-range rainbow marker; lane 2, synthesized EP; lane 3, protein expressed by the pPICZαA vector; lane 4-9, cells containing EP expression vector after induction for 0 h (no methanol induction), 1, 2, 3, 4 and 5 days.

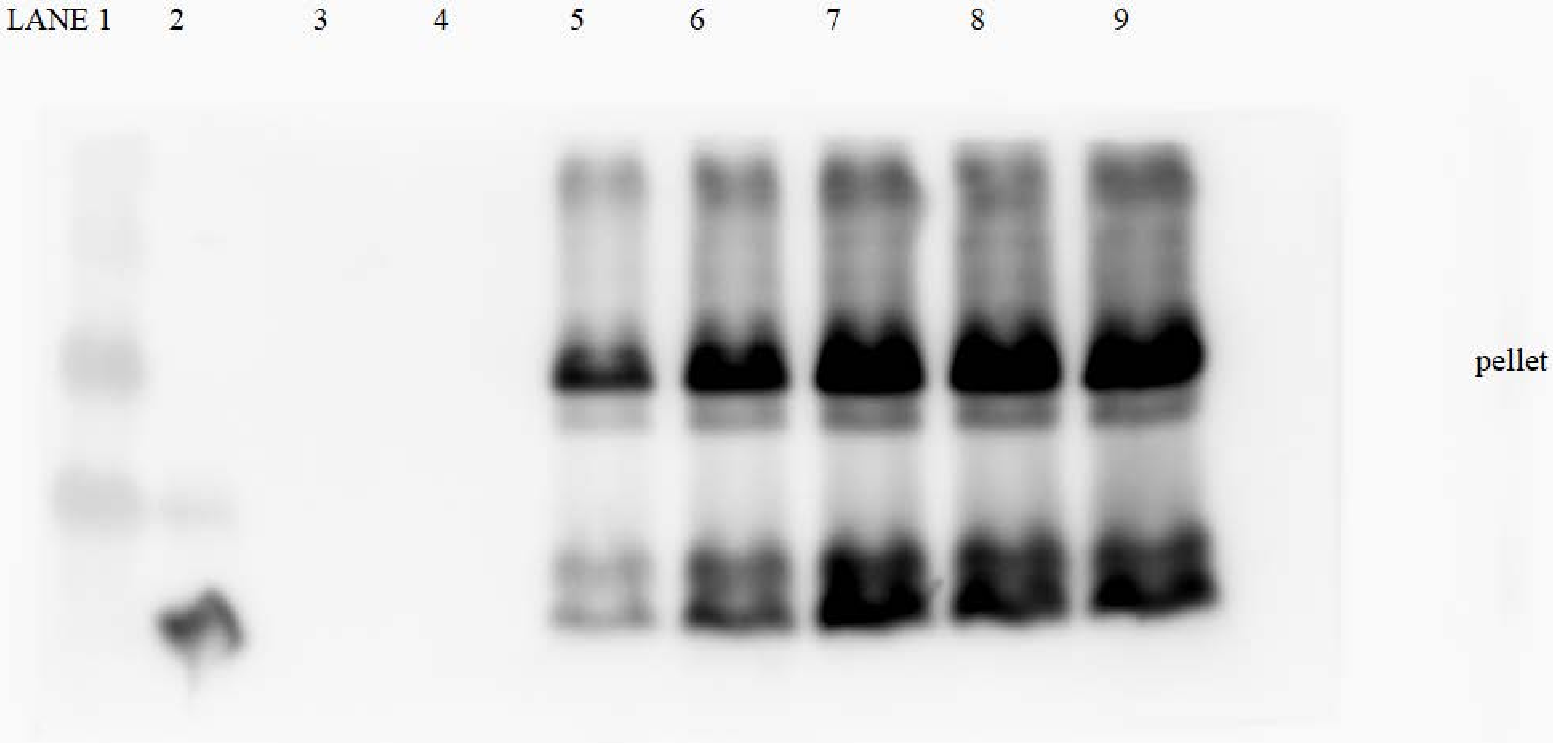

Figure 2c was generated from thta original image

Cells were harvested and total protein from supernatant and pellet were analyzed by SDS-PAGE and western blotting. Lane 1, low-range rainbow marker; lane 2, synthesized EP; lane 3, protein expressed by the pPICZαA vector; lane 4-9, cells containing EP expression vector after induction for 0 h (no methanol induction), 1, 2, 3, 4 and 5 days.

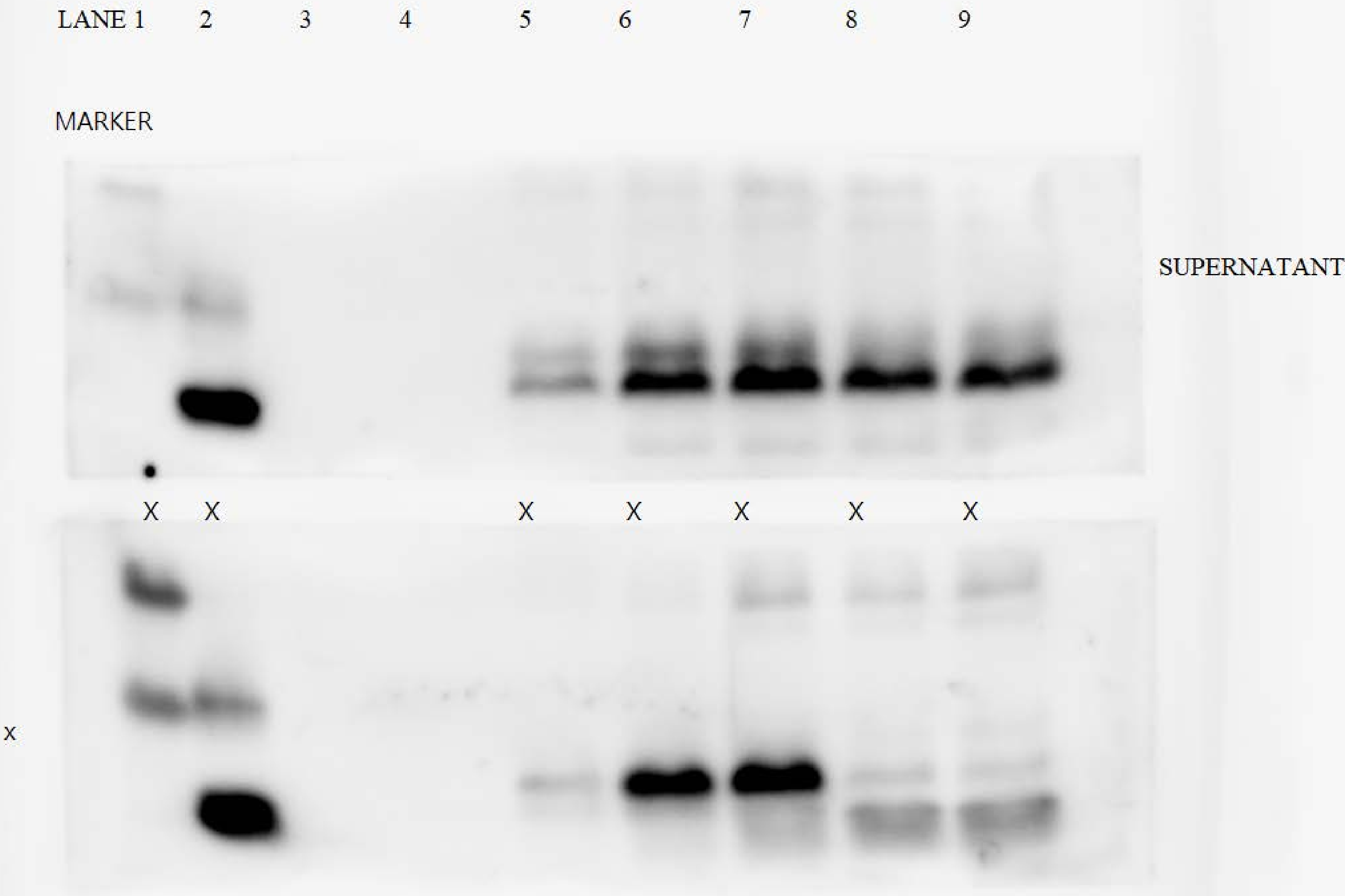

Figure 2c was
generated from that
original image
